# Identification of a PH domain-containing protein which is localized to crystalloid bodies of *Plasmodium* ookinetes

**DOI:** 10.1186/s12936-018-2617-6

**Published:** 2018-12-13

**Authors:** Rachaneeporn Jenwithisuk, Niwat Kangwanrangsan, Mayumi Tachibana, Amporn Thongkukiatkul, Hitoshi Otsuki, Jetsumon Sattabongkot, Takafumi Tsuboi, Motomi Torii, Tomoko Ishino

**Affiliations:** 1 Division of Molecular Parasitology, Proteo-Science Center, Ehime University, Toon, Ehime 791-0295 Japan; 2 Mahidol Vivax Research Unit, Faculty of Tropical Medicine, Mahidol University, Bangkok, 10400 Thailand; 3 Department of Pathobiology, Faculty of Science, Mahidol University, Bangkok, 10400 Thailand; 4 Department of Biology, Faculty of Science, Burapha University, Chonburi, 20131 Thailand; 5 Division of Medical Zoology, Faculty of Medicine, Tottori University, Yonago, Tottori 683-8503 Japan; 6 Division of Malaria Research, Proteo-Science Center, Ehime University, Matsuyama, Ehime 790-8577 Japan

**Keywords:** *Plasmodium yoelii*, Ookinete, Zygote, Crystalloid body, PH domain

## Abstract

**Background:**

For the success of the malaria control and eradication programme it is essential to reduce parasite transmission by mosquito vectors. In the midguts of mosquitoes fed with parasite-infected blood, sexual-stage parasites fertilize to develop into motile ookinetes that traverse midgut epithelial cells and reside adjacent the basal lamina. Therefore, the ookinete is a promising target of transmission-blocking vaccines to break the parasite lifecycle in mosquito vectors. However, the molecular mechanisms of ookinete formation and invasion of epithelial cells have not been fully elucidated. A unique structure called the crystalloid body has been identified in the ookinete cytoplasm by electron microscopy, but its biological functions remain unclear.

**Methods:**

A recombinant protein of a novel molecule, designated as crystalloid body specific PH domain-containing protein of *Plasmodium yoelii* (*Py*CryPH), was synthesized using a wheat germ cell-free system. Specific rabbit antibodies against *Py*CryPH were obtained to characterize the expression and localization of *Py*CryPH during sexual-stage parasite development. In addition, *Py*CryPH knockout parasites were generated by targeted gene disruption to examine *Py*CryPH function in mosquito-stage parasite development.

**Results:**

Western blot and immunofluorescence assays using specific antibodies showed that *Py*CryPH is specifically expressed in zygotes and ookinetes. By immunoelectron microscopy it was demonstrated that *Py*CryPH is localized within crystalloid bodies. Parasites with a disrupted *PyCryPH* gene developed normally into ookinetes and formed oocysts on the basal lamina of midguts. In addition, the number of sporozoites residing in salivary glands was comparable to that of wild-type parasites.

**Conclusions:**

CryPH, containing a signal peptide and PH domain, is predominantly expressed in zygotes and ookinetes and is localized to crystalloid bodies in *P. yoelii*. CryPH accumulates in vesicle-like structures prior to the appearance of typical crystalloid bodies. Unlike other known crystalloid body localized proteins, CryPH does not appear to have a multiple domain architecture characteristic of the LAP/CCp family proteins. Although CryPH is highly conserved among *Plasmodium*, *Babesia*, *Theileria*, and *Cryptosporidium*, *Py*CryPH is dispensable for the development of invasive ookinetes and sporozoites in mosquito bodies.

**Electronic supplementary material:**

The online version of this article (10.1186/s12936-018-2617-6) contains supplementary material, which is available to authorized users.

## Background

Malaria is caused by the transmission of *Plasmodium* spp. to mammalian hosts by *Anopheles* mosquitoes. Malaria transmission begins with the uptake of infected blood containing sexual-stage gametocyte parasites, which leads to rapid gametogenesis followed by fertilization to form zygotes. The parasites then transform into motile ookinetes that migrate through the midgut epithelium to the basal lamina. At this site the ookinetes transform into oocysts, within which thousands of sporozoites develop [1]. Upon their release into haemolymph, sporozoites invade salivary glands, to be injected into mammalian hosts together with saliva [2, 3]. Ookinete formation and its traverse of midgut epithelium are indispensable events in the series of steps for parasite transmission via mosquito vectors. Two infectious forms of *Plasmodium*, merozoites and sporozoites, form a parasitophorous vacuole (PV) during the invasion of target cells and subsequent maturation within the PV space. In contrast, ookinetes traverse midgut epithelial cells without forming a PV, and reside extracellularly adjacent the basal lamina [4, 5]. Ookinetes are morphologically distinct from the other two invasive forms, merozoites and sporozoites, in that they lack rhoptries and contain crystalloid bodies in their cytosol [6–8]. Crystalloid bodies have been described as spherical organelles with a honeycomb-like or viral inclusion-like structure [9–12]. Crystalloid bodies are observed in numerous parasites in the phylum Apicomplexa, such as *Plasmodium* [10, 11, 13, 14], *Haemoproteus* [11], *Leucocytozoon* [11, 15, 16], *Hepatozoon* [17], *Haemolivia* [18], *Isospora* [19–21], *Hammondia* [22], *Cryptosporidium* [23, 24], and *Eleutheroschizon* [25]. Although crystalloid bodies have been described for their characteristic structure, their formation and function remain largely unknown. Members of the LCCL lectin adhesive-like protein (LAP) family, which share a conserved architecture of multiple predicted adhesive domains including the LCCL (*Limulus* coagulation factor C, Coch-5b2 and Lgl1) domain, have been demonstrated to localize to crystalloid bodies of *Plasmodium berghei* ookinetes [14, 26, 27]. These molecules are categorized as the CCp family in *Plasmodium falciparum* and were shown to form multi-protein complexes in sexual-stage parasites [28–32]. Disruption of *LAP/CCp* genes, either individually or in pairs, give rise to similar loss-of-function phenotypes; specifically, a failure of oocyst development to produce infective sporozoites (see Table 1) [14, 29, 30, 33–37]. These data suggested that crystalloid bodies play an important role for subsequent sporozoite formation and/or its invasive ability, but further studies are needed to understand the specific functions of crystalloid bodies. In the course of screening for novel secreted or membrane proteins of sexual-stage parasites based on microarray data of *P. falciparum* stage-specific gene expression [38] and *P. berghei* AP2-G2 KO gametocyte gene expression [39], a protein with a pleckstrin homology (PH) domain, which is localized in the crystalloid bodies of ookinetes, was identified. In this study, this protein, designated as CryPH, was characterized in the rodent malaria parasite line *Plasmodium yoelii* 17XNL (*Py*XNL). In addition, the role of *Py*CryPH during parasite development in mosquito bodies was examined by generating *PyCryPH* gene-disrupted parasites.

**Table 1:** Summary of CCp/LAP expression and phenotype in *Plasmodium*

	*P. berghei/P. yoelii*			*P. falciparum*	
	Accession no.	Subcellular localization	Function or KO phenotype	Subcellular localization	Function or KO phenotype
CryPH	PBANKA_0704900	Crystalloid body	CryPH is dispensable for ookinete, oocyst, sporozoite formation	ND	ND
PfCCp1/PbLAP2	PBANKA_1300700	Gametocyte cytoplasm and crystalloid body in ookinetes [26]	LAP2 knockout parasites form oocysts in the normal number, but fail to produce sporozoites inside [35]	Female gametocyte surface (punctate pattern) [31]	
PfCCp2/PbLAP4	PBANKA_1319500	Crystalloid body in developing ookinetes [27]	LAP4 is essential for oocyst maturation and sporozoite formation [35]	Female gametocyte surface (punctate pattern) [31]	In CCp2 deficient parasites, oocyst formation is normal but sporozoites were unable to invade salivary glands [29]
PfCCp3/PbLAP1 (PbSR)	PBANKA_1035200	Crystalloid body in developing ookinetes [14]	PbSR is involved in crystalloid formation. It is dispensable for the oocyst formation but essential for sporozoite formation inside [14, 33]	Female gametocyte surface (punctate pattern) [31]	CCp3 deficient mutant lacks CCp1 and CCp2. Sporozoites form normally in oocysts, however they lost abilities of release into hemocoel and invasion of salivary glands [29]
PfCCp4/PbLAP6	PBANKA_0417600	Crystalloid body in developing ookinetes [27]	LAP6 is essential for oocyst maturation and sporozoite formation [35]	Female gametocyte surface (homogenous expression) [31] Co-localized with Pfs230	CCp4 is dispensable for parasite development in the mosquito vector [31]
PfCCp5/PbLAP3	PBANKA_0204500	Gametocyte cytoplasm and crystalloid body in ookinetes [26]	LAP3 is essential for crystalloid body formation, and for oocyst maturation to produce sporozoites [67]	Female gametocytes (at the pole) [31]	
PfFNPA/PbLAP5	PBANKA_1315300	Crystalloid body in developing ookinetes [27]	LAP5 knockout parasites form oocysts in the normal number, but fail to produce sporozoites inside [36]	Female gametocytes (at the pole) [31]	

Carter et al. [14], Saeed et al. [26, 27], Pradel et al. [29], Scholz et al. [31], Claudianos et al. [33], Raine et al. [35], Ecker et al. [36], Saeed et al. [67] 2015

*ND* not done

## Methods

### Parasites and mosquitoes

Cryopreserved *Py*XNL infected erythrocytes were intraperitoneally injected into 4–6-week-old ICR female mice (CLEA Japan, Tokyo, Japan) to obtain asexual- and sexual-stage parasites. Mice were kept in a room with a temperature of 24 °C under a 12 h light/12 h dark cycle. For mosquito feeding experiments, *Py*XNL-infected mouse erythrocytes diluted with RPMI 1640 (Wako Pure Chemical, Osaka, Japan) were fed to *Anopheles stephensi* SDA500 mosquitoes through a membrane-feeding apparatus. Fully engorged mosquitoes were selected and kept at 24 °C until dissection. On days 10 and 17 post-feeding, midguts and salivary glands were collected by dissection to count the numbers of oocysts and sporozoites, respectively. All animal experimental protocols were approved by the Institutional Animal Care and Use Committee of Ehime University, and the experiments were conducted according to the Ethical Guidelines for Animal Experiments of Ehime University.

### In silico screening

To identify secreted or membrane-anchored proteins in sexual-stage parasites, candidate genes were selected using information within the PlasmoDB database [40] as follows: (1) identification of transcripts whose levels in *P. falciparum* gametocytes are > 3-fold greater than in *P. falciparum* sporozoites, and are not abundant (below the 50th percentile) in *P. falciparum* schizonts [38]; (2) possession of a secondary structure containing a signal peptide and/or transmembrane domain; (3) orthologues are conserved among *Plasmodium* spp.; and (4) *P. berghei* genes transcripts in wild-type gametocytes are > 100-fold more than those in AP2-G2 KO gametocytes [39]. Sixty-two genes were selected by the first two criteria, and of these 32 genes were selected as candidates expressed in the sexual-stage parasites as predicted secreted- or membrane anchored- proteins.

#### Recombinant proteins and antiserum production

Recombinant *Py*CryPH (rCryPH) was produced using the wheat germ cell-free protein synthesis system (CellFree Sciences, Matsuyama, Japan) as described [41, 42]. Briefly, a DNA fragment encoding *Py*CryPH (PY17X_0705200) without the N-terminal signal peptide (amino acids 19–287 of CryPH) was amplified from *Py*XNL genomic DNA using Phusion high-fidelity DNA polymerase (New England BioLabs, Ipswich, MA, USA) with a primer pair 5′-ctcgagCATAAAAATTTCTCAAGAAGAAATAATTAC-3′ and 5′-ggatccTCATTTGAGAGAAATATTTGGATTGC-3′. The amplified *PyCryPH* DNA fragment was cloned into the pEU-E01-HisGST (TEV)-N2 vector (CellFree Sciences) between the XhoI and BamHI recognition sites. The DNA sequence of the insert was confirmed using an ABI PRISM 3100 Genetic Analyzer and a BigDye Terminator v1.1 Cycle Sequencing kit (Applied Biosystems, Foster City, CA, USA). *Py*CryPH GST fusion recombinant protein was expressed using the wheat germ cell-free protein synthesis system (CellFree Sciences), and then tag-free rCryPH was purified by on-column cleavage using AcTEV protease (Invitrogen, Carlsbad, CA, USA) and the glutathione-Sepharose 4B column (GE Healthcare, Camarillo, CA, USA). Protein synthesis was confirmed by separation on SDS-PAGE under reducing condition and visualized with Coomassie Brilliant Blue protein staining. Protein concentration was determined using a Bradford protein assay kit (Bio-Rad Laboratories, Hercules, CA, USA).

To generate antibodies against CryPH, a Japanese white rabbit was immunized subcutaneously with 250 μg rCryPH with Freund’s complete adjuvant followed by two booster immunizations with 250 μg rCryPH with Freund’s incomplete adjuvant. All immunizations were done at 3-week intervals, and antisera were collected 14 days after the last immunization (Kitayama labes Co. Ltd. Ina, Japan). Anti-CryPH rabbit antibodies were affinity purified using a HiTrap NHS-activated HP column (GE Healthcare) coupled to rCryPH.

### Construction of GFP-tagged CryPH expressing parasites and CryPH disrupted parasites

To generate GFP-tagged CryPH expressing parasites (CryPH-GFP), the *PyCryPH/PY17X_0705200* locus was replaced by double-crossover homologous recombination method with the coding region of CryPH fused in-frame with a GFP coding sequence at the C-terminus. As shown in Additional file 1: Figure S1a, two homologous recombination cassettes—the C-terminal region of CryPH coding sequence (CryPH-C) and the 3′-UTR of CryPH (CryPH-3′)—were sub-cloned into the pGFPDT-B12 vector, which was modified from pPbDT3U-B12 [43] to contain a GFP coding sequence. An 807 bp DNA fragment corresponding to CryPH-C and an 849 bp fragment corresponding to CryPH-3′ were PCR amplified from *Py*XNL genomic DNA using the following primers: CryPH.F1 (5′-ctcgagCATAAAAATTTCTCAAGAAGAAATAATTAC-3′) plus CryPH.NheI.R (5′-gctagcTTTGAGAGAAATATTTGGATTGC-3′); and CryPH.3UTR.SalI.F (5′-gtcgacCGAATAGGGAAAAAAAAATCTCC-3′) plus CryPH.3UTR.XhoI.R (5′-ctcgagGGGTATCTGACTTTATATTGAGC-3′). For transgenic parasite selection, human dihydrofolate reductase gene (hDHFR) coding sequence was introduced into the vector by Gateway technology (Invitrogen) using pHDEF-1-mh-R12 [44].

The same strategy as described above was applied to generate *CryPH*-disrupted parasites (ΔCryPH). Instead of CryPH-C, the 5′-UTR of CryPH (CryPH-5′) was used as one of the cassettes for homologous recombination (Additional file 1: Figure S1b). A 965 bp fragment corresponding to CryPH-5′ was amplified by PCR with the following primers: CryPH.5UTR.XhoI.F (5′-ctcgagGGAATAGCTATGCATATATGCG-3′) and CryPH.5UTR.BamHI.R (5′-ggatccCAATTTACAATAACAACAAAATATGG-3′). The fragments CryPH-5′ and CryPH-3′ were cloned into the pPbDT3U-B12 transgenic vector. The drug-resistant cassette was inserted as described above. To prepare control parasites (CryPH-cont), the same drug-resistant cassette was integrated just after the *PyCryPH* gene by homologous recombination (Additional file 1: Figure S1c). Two homologous recombination cassettes CryPH-C, conjugated to the 3′UTR of PbDHFR/ts, and CryPH-3′ were cloned into the pPbDT3U-B12 vector as described above.

Transfection of *Py*XNL parasites (PyWT) was performed as described with some modification [45, 46]. Briefly, PyWT schizont-infected erythrocytes were enriched by density gradient centrifugation and transfected with linearized plasmid DNA using the Nucleofector 2b (Lonza Japan, Tokyo, Japan) with human T cell solution under program U-33. After electroporation, infected erythrocytes were intravenously injected into 4-week-old ICR female mice. Drug selection was initiated 24 h after inoculation by adding 70 μg/mL pyrimethamine to the drinking water. The integration of the target DNA fragment was detected by genotyping PCR, using attB1-link.AvF (5′-CTAGACAAGTTTGTACAAAAAAGCAGG-3′) and PY00516.R (5′-TTGATAACATGCTATCATGTCG-3′). The wild-type locus was detected by PCR using PyCryPH.F2 (5′-CCGATATACCAAAGCATTCAAC-3′) and CryPH.3UTR.XhoI.R (5′-ctcgagGGGTATCTGACTTTATATTGAGC-3′). Non-linearized plasmid (episomal form) was detected by PCR using PyCryPH.3UTR.F1 (5′-GCACACAAATATGTGTATCAATG-3′) and PbDT3U.R1 (5′-ACAGTTATAAATACAATCAATTGG-3′). A clone of CryPH-GFP, two independent clones of ΔCryPH, and a clone of CryPH-cont were isolated by limiting dilution.

### In vitro ookinete culture

For in vitro ookinete culture, cryopreserved *Py*XNL-infected blood was intraperitoneally injected into ICR mice pretreated with 1.2 mg phenylhydrazine to induce reticulocyte production. Infected blood with a high number of gametocytes collected from these mice was diluted fourfold with suspended animation medium (9 mM glucose, 8 mM Tris-base, 138 mM sodium chloride, pH7.3) pre-warmed at 37 °C. After passing through a CF11 column to deplete white blood cells, the infected erythrocytes were resuspended with 20 volumes of ookinete culture medium (RPMI 1640 medium containing 20% heat inactivated fetal calf serum, 0.367 mM hypoxanthine, 25 mM 4-(2-hydroxyethyl)-1-piperazineethanesulfonic acid (HEPES) and 5 IU/mL heparin, pH 8.3), and incubated at 24 °C [44]. At 0, 1, 4, and 14–18 h after incubation, equal volumes of cultured parasites were collected for further analyses. To enrich sexual-stage parasites, density gradient centrifugation using 14% (w/v) histodenz (Sigma-Aldrich Company Ltd, St. Louis, MO, USA) was performed [47]. The parasites were collected on the interface and washed twice with ice-cold PBS containing protease inhibitors (PBS-PI; Roche Applied Science, Penzberg, Germany). Giemsa-stained smears on glass slides were used to assess the stage and purity of cultured parasites. The number of parasites obtained after purification were counted in a hemocytometer.

### Western blotting analysis

Parasite lysates (gametocytes, ookinetes, and schizonts) were prepared by repeated freeze–thaw cycles in 1% Triton X-100 in PBS-PI and heated in SDS-PAGE loading buffer in the absence or presence of 4% ß-mercaptoethanol at 95 °C for 5 min. Sporozoites were collected from midguts of infected mosquitoes and heated in SDS-PAGE loading buffer containing 4% ß-mercaptoethanol. Equivalent numbers of parasites per lane were subjected to electrophoresis in a 5–20% gradient polyacrylamide gel (ATTO, Tokyo, Japan) and then transferred to a polyvinylidene fluoride membrane (BioRad). Membranes were blocked in Blocking One (Nacalai tesque, Kyoto, Japan) overnight at 4 °C, followed by immunostaining with anti-CryPH antibodies (1: 2000) for 1 h at room temperature. Anti-Pys25 mouse monoclonal antibodies (mAb) were used as a zygote/ookinete marker [48]. Anti-RAMA antiserum (1: 2000) was used as a positive marker for schizonts and sporozoites [49]. Membranes were then stained with secondary antibodies (goat anti-rabbit IgG or goat anti-mouse IgG) conjugated to horseradish peroxidase (HRP; Thermo Fisher Scientific, Waltham, MA, USA; 1:20,000) for 30 min at room temperature. Chemiluminescent detection was performed by adding Immobilon Western Chemiluminescence HRP substrate (Merck Millipore, Darmstadt, Germany).

### Immunofluorescence assay (IFA)

Smears of cultured *Py*XNL parasites were fixed on glass slides with ice-cold acetone for 5 min and blocked with PBS containing 5% nonfat milk at 37 °C for 30 min. They were then incubated with rabbit anti-CryPH antibodies (1: 1000), rabbit anti-GFP antibody (1: 500, Clontech, Mountain View, CA, USA) and mouse anti-Pys25 mAb (1: 20,000) at 37 °C for 1 h, and thereafter with Alexa Fluor 488-goat anti-rabbit IgG antibody and Alexa Fluor 546-goat anti-mouse IgG antibody (Invitrogen) as secondary antibodies (1:500 dilution) at 37 °C for 30 min, together with 1 μg/mL 4′,6-diamidino-2-phenylindole (DAPI). After mounting in ProLong Gold antifade reagent (Invitrogen), samples were observed with an inverted fluorescence microscope (Axio observer z1, Carl Zeiss, Oberkochen, Germany), and images were taken using AxioVision software (Carl Zeiss).

### Immunoelectron microscopy (IEM)

In vitro cultured *Py*XNL zygotes/ookinetes were fixed for 30 min on ice in a mixture of 1% paraformaldehyde and 0.2% glutaraldehyde in 1× HEPES buffer (pH 7.05), then dehydrated and embedded in LR white resin (Polyscience Inc., Warrington, PA, USA). Ultrathin sections were blocked for 30 min in PBS containing 5% nonfat dry milk and 0.01% Tween 20 (PBS-MT), followed by overnight incubation with specific antibodies (1:50) in PBS-MT. After washing by PBS containing 10% BlockAce (DS-Phama Co, Osaka, Japan) and 0.01% Tween 20, samples were then incubated for 1 h in PBS-MT containing goat anti-rabbit IgG conjugated to 15 nm gold particles (1:20, BBI International, Minneapolis, MN, USA) [50]. The grids were stained with 2% uranyl acetate in 50% methanol and lead citrate. Samples were examined with a transmission electron microscope (JEM-1230; JEOL, Tokyo, Japan).

### Infectivity assay

Parasite infected erythrocytes (1 × 10^5^) or sporozoites (1 × 10^4^) collected from salivary glands of CryPH-cont or ∆CryPH infected mosquitoes, were inoculated into 4-week-old female ICR mice. Parasitaemias of infected mice were determined daily by Giemsa staining.

## Results

### Identification of pleckstrin homology domain-containing sexual-stage specific proteins conserved in *Plasmodium* spp.

**Fig. 1:**
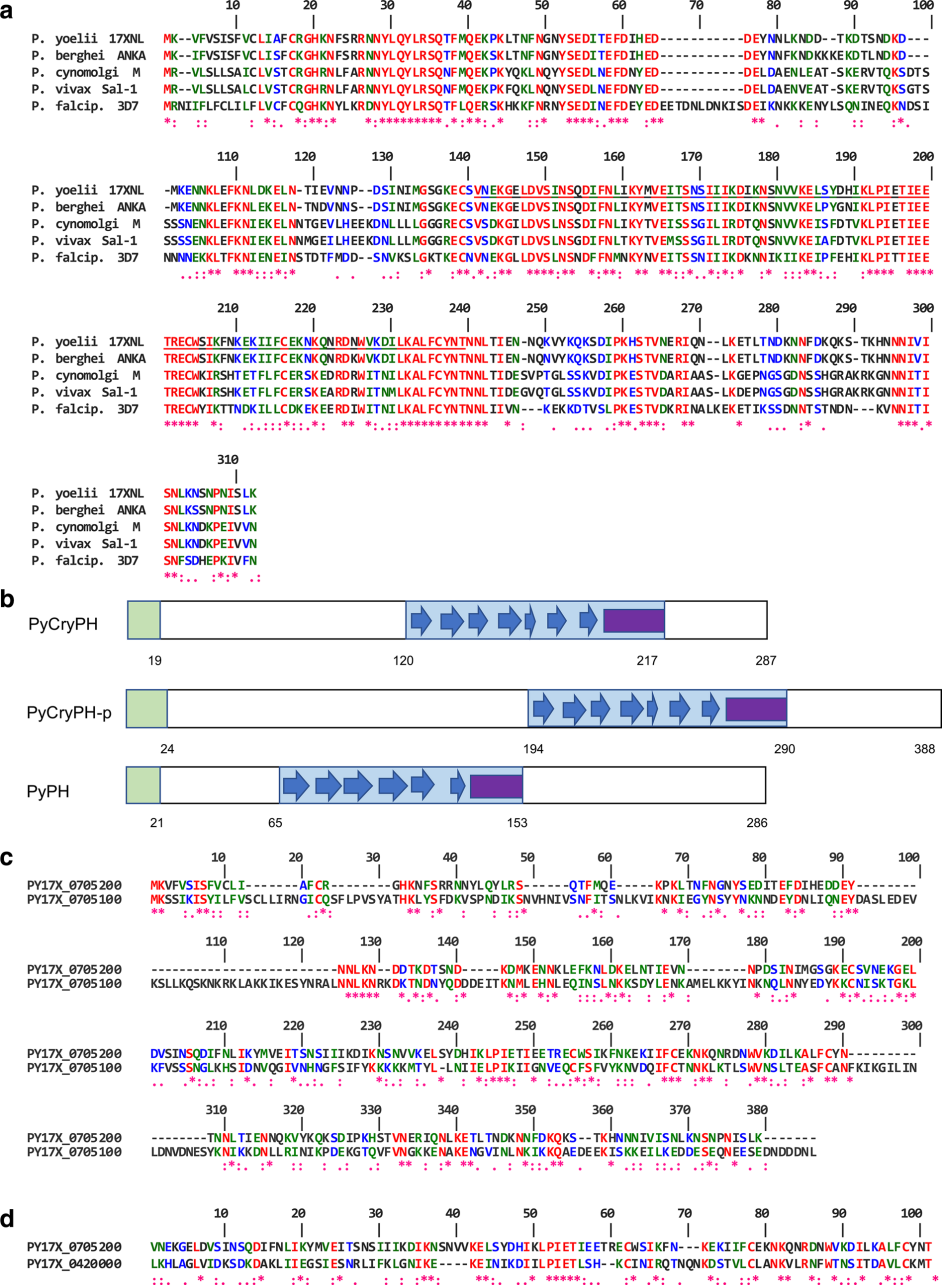
Sequence alignment and predicted secondary structure of CryPH compared with its orthologues and PH domain family proteins. **a** Alignment of amino acid sequences of CryPH orthologues in *Plasmodium*. Amino acid sequences of *Plasmodium* CryPH (PY17X_0705200, PBANKA_0704900, PcyM_0507700, PVX_089075, PF3D7_0825700) are aligned using CLUSTALW program (https://npsa-prabi.ibcp.fr/cgi-bin/npsa_automat.pl?page=npsa_clustalw.html). **b** Schematic representation of the secondary structure of *Py*CryPH, *Py*CryPH-p (PY17X_0705100), and *Py*PH (PY17X_0420000). Green and blue squares indicate the N-terminal signal peptide and PH domain. Blue arrows and purple square indicate ß-sheets and α-helix. Numbers indicate amino acid positions from the first methionine. **c** Amino acid sequence comparison between *Py*CryPH and *Py*CryPH-p. Twenty percent of residues including all four cysteine residues are conserved between *Py*CryPH and *Py*CrePH-p. **d** Amino acid sequence alignment between PH domains of *Py*CryPH and *Py*PH. “*”, “:”, and “.” indicate amino acid residues with full identity, strong similarity, and weak similarity, respectively

To identify novel secreted or membrane-anchored proteins in sexual-stage parasites (gametes, zygotes, and ookinetes) in silico screening of the PlasmoDB databases was used to select 32 genes as candidates, as described in the methods section. This data set contains known surface/secreted proteins in gametocytes and ookinetes, such as P47 [51], two CPW-WPC proteins [44, 52], and four putative secreted ookinete proteins (PSOP) [36]; thus assuring that our criteria were reasonable. In this study, an uncharacterized protein containing 287 amino acid residues with an N-terminal signal peptide (PY17X_0705200 in *P. yoelii*) was focused, whose transcription was apparently regulated by the sexual stage-specific transcription factors, AP2-G, AP2-G2, and AP2-O [53]. BLAST and synteny analyses [40] demonstrate that all *Plasmodium* spp. contain orthologous genes to PY17X_0705200, which are highly conserved in amino acid sequences (Fig. 1a); about 37% and 20% of amino acid residues are identical and strongly similar, respectively, among *Plasmodium* spp. The domain search algorithm SMART [54] and structure prediction algorithm (Phyre2) [55] revealed that PY17X_0705200 contains a pleckstrin homology domain (PH domain), consisting of a ß-barrel of seven anti-parallel ß-sheets and an amphiphilic α-helix (Fig. 1b). As it contains a PH domain and localizes to crystalloid bodies, demonstrated below, this protein was designated as *Py*CryPH. By BLAST search using EupathDB (the Eukaryotic Pathogen Genomics Database) [56], predicted orthologous genes were found in the order Piroplasmida (*Babesia* and *Theileria*) and *Cryptosporidium,* with conserved features of an N-terminal signal peptide and a PH domain. A possible homologue was also found in *Toxoplasma*, with relatively high amino acid sequence similarity despite the lack of an N-terminal signal peptide. Recent strand-specific RNA-seq analysis in *Toxoplasma gondii* raised the possibility that its translation starts from the internal methionine (at aa position 44 in TGME49_219160) that is immediately followed by a hydrophobic region that is robustly predicted to be a signal peptide sequence (SignalP; cbs.dtu.dk/services/SignalP/) [57]. Thus, the possibility should be considered that either the gene predictions in the *Toxoplasma* orthologues are in error or that translation is initiated at the downstream methionine. The alignment of amino acid sequences demonstrates that the sequence similarity is higher in the PH domain region and all four cysteine residues are conserved among CryPH orthologues (Additional file 1: Figure S2).

BLAST search and Phyre2 analysis revealed a gene adjacent to PY17X_0705200 in a tail-to-tail orientation which encodes a predicted paralogous protein containing a signal peptide and PH domain (PY17X_0705100, designated as *PyCryPH*-*p*; Fig. 1b) and 20% identical amino acid residues (Fig. 1c). CryPH and CryPH-p orthologous genes are also adjacent in *Babesia*, *Theileria*, and *Cryptosporidium.* In addition, it was recently reported that a secretory protein with a PH domain is expressed in zygotes/ookinetes and localizes to the surface membrane (PY17X_0420000) [58]. The identity of amino acid residues in the PH domain of both proteins is about 25% and cysteine residues are conserved (Fig. 1d), although overall identity is low (17%). The expression pattern of these three genes are similar; specifically, dominantly transcribed in female gametocytes and AP2-G2 dependent, which is also confirmed by the fact that all are selected by our screening criteria. Taken together, these pieces of evidence suggest that CryPH, CryPH-p, and PH belong to a PH domain-containing protein family having sexual stage expression. Molecular phylogenetic analysis (Additional file 1: Figure S3) suggests that CryPH, CryPH-p, and PH genes originated before genera within the phylum Apicomplexa diverged.

### CryPH is expressed in zygotes and ookinetes

**Fig. 2:**
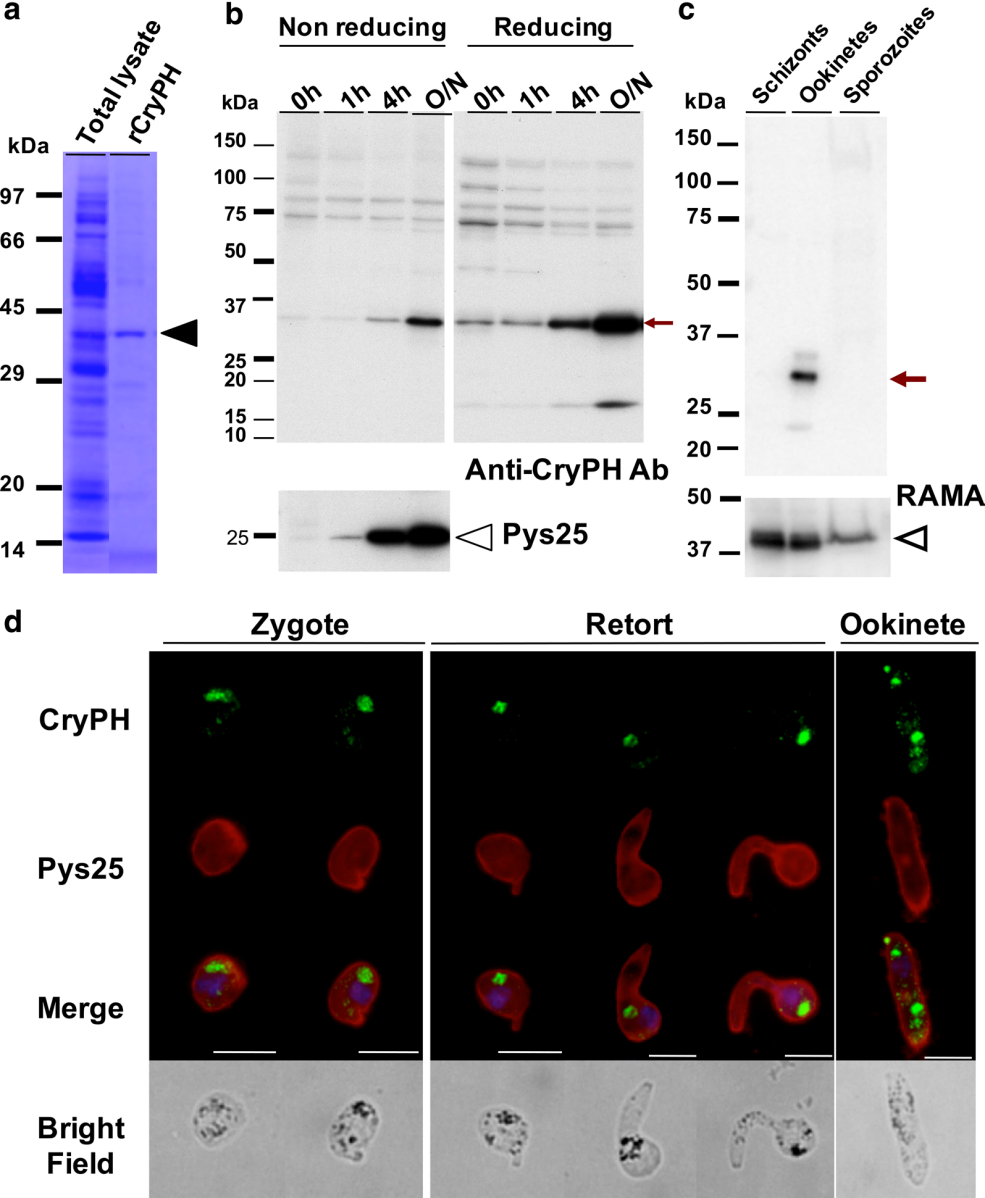
*Py*CryPH is expressed in zygotes and ookinetes. **a** Expression of recombinant CryPH using the wheat germ cell-free protein expression system. Predicted size of the recombinant CryPH is indicated by an arrowhead. **b** Expression profile of CryPH examined by Western blotting analysis using extracts from in vitro ookinete culture. The same volumes of cultured parasites were taken at the times indicated above. Affinity purified anti-CryPH antibodies were used both under non-reducing (left panel) or reducing (right panel) conditions. The representative stages of parasites at each time point were as follows: 0 h, gametocytes and gametes; 1 h, zygotes; 4 h, retorts; and overnight incubation (O/N), mature ookinetes. The arrow indicates the bands corresponding to CryPH. The same filter was probed with anti-Pys25 antibodies, a zygote/ookinete marker (Lower Panel). Open arrowhead indicates Pys25. **c** Expression comparison of CryPH in all infective stages. Protein lysates of schizonts, ookinetes, and oocyst-derived sporozoites (1 × 10^5^) were separated by SDS-PAGE and CryPH expression, indicated by an arrow, was detected by Western blotting using anti-PyCryPH antibodies. Protein loading was assessed by re-probing with anti-RAMA antiserum (lower panel, arrow head). **d** Localization analysis of CryPH using immunofluorescence assay. CryPH was detected as clear circular spots in the parasite cytosol, and the signal intensity increased during ookinete development. Zygotes and ookinetes (retorts and mature ookinetes) cultured in vitro were fixed with acetone for immunofluorescence assays. CryPH signals were associated with malaria pigments. Merge, merged image of CryPH (green), Pys25 (red), and nuclei stained by DAPI (blue). Bars, 5 µm

To raise specific rabbit antibodies against CryPH of *P. yoelii*, a recombinant protein corresponding to full-length CryPH lacking the N-terminal signal peptide (rCryPH) was produced by a wheat germ cell-free protein synthesis system (Fig. 2a). To evaluate the reactivity of anti-CryPH antibodies against native CryPH in the parasite lysate, Western blotting was performed using cultured zygotes/ookinetes of *Py*XNL parasites. Anti-CryPH antibodies recognize a protein in parasite lysates as a major band of approximately 35 kDa under both non-reducing and reducing conditions, corresponding to a calculated molecular weight of CryPH (33.7 kDa; Fig. 2b, the lanes indicating overnight (O/N) culturing). The CryPH band intensity was faint before the start of parasite culturing (0 h) when gametocytes were predominant but increased with ookinete maturation. CryPH expression occurs almost simultaneously or slightly later than that of Pys25, a major surface molecule of zygotes and ookinetes. To determine CryPH protein profiling through the lifecycle, Western blotting was performed with all infective stage antigens; namely, schizonts (containing merozoites), ookinetes, and sporozoites derived from oocysts. CryPH expression was detected specifically in ookinetes (Fig. 2c).

To investigate CryPH localization in zygotes and ookinetes, IFA was performed using anti-CryPH antibodies. CryPH was initially detected as a diffuse or scattered pattern in the cytoplasm of zygotes. As parasites elongated to form mature ookinetes, the CryPH signal condensed as clear round shapes in the cytoplasm that overlapped with malaria pigments detected in the bright field images (Fig. 2d).

**Fig. 3:**
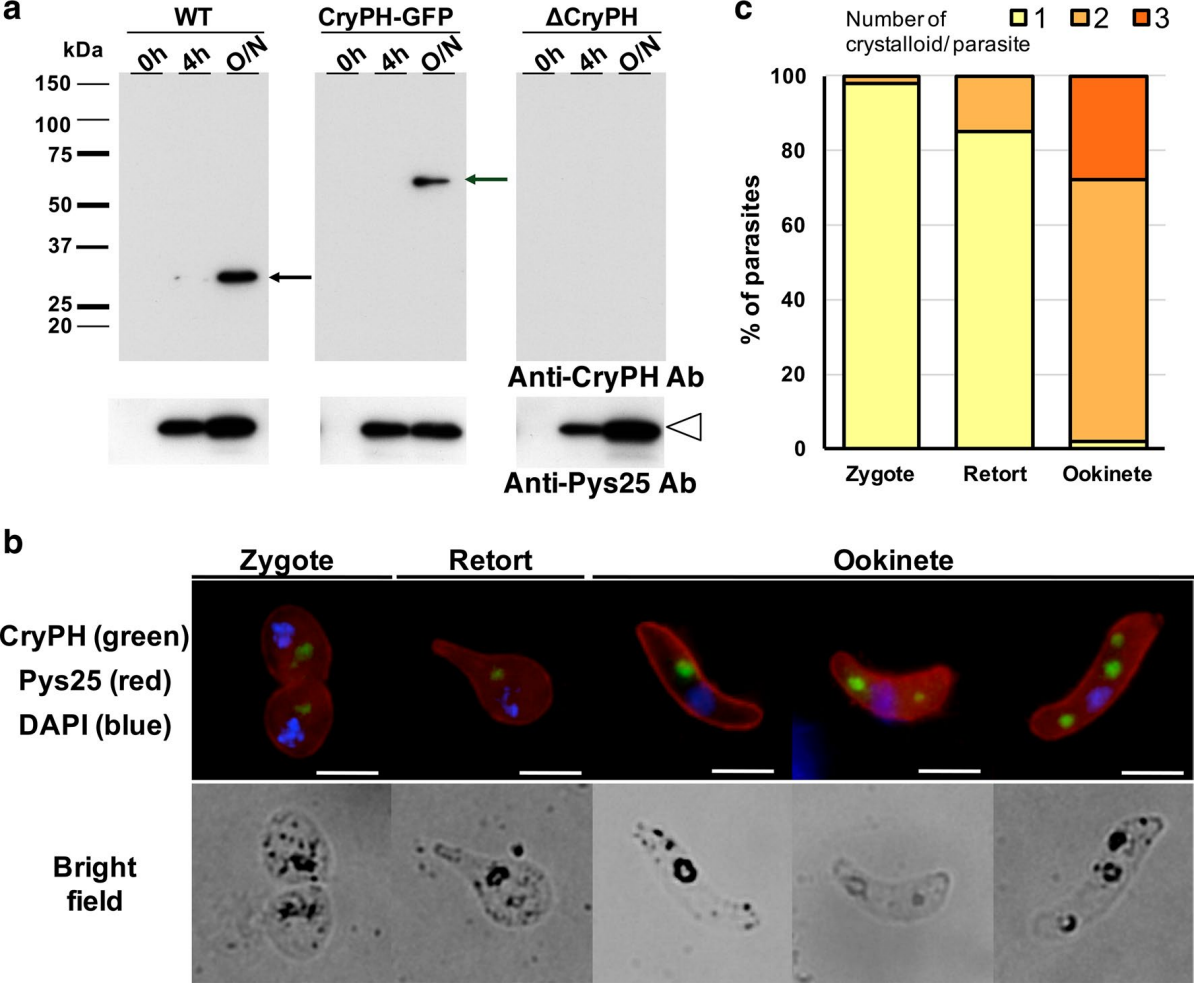
Construction of CryPH-GFP expressing or *CryPH* gene depleted transgenic parasites. **a** Western blot analysis for confirmation of gene modification in isolated parasites. Wild-type (WT) or genetically modified (CryPH-GFP or ∆CryPH) *Py*XNL parasite lines cultured in vitro were collected at indicated time points. These parasites were analyzed by Western blot using anti-CryPH antibodies and anti-Pys25 antibodies under non-reducing conditions. Left panels, a clear protein signal of the size comparable to that of native *Py*CryPH (indicated by a black arrow) was detected in WT ookinete lysate. Middle panels, one major band of about 60 kDa corresponding to the predicted size of CryPH-GFP (indicated by a green arrow) was revealed in CryPH-GFP expressing transgenic ookinetes. Note that there was no signal of the size of native *Py*CryPH. Right panels, disappearance of *Py*CryPH signal in ookinetes with genetically disrupted *PyCryPH* showed that CryPH expression was completely depleted. In contrast, Pys25 signal intensity was increased during culturing, suggesting that ∆CryPH ookinetes formed normally (lower panel, indicated by an open arrow head). **b** Detection of CryPH-GFP by immunofluorescence analysis. Cultured CryPH-GFP parasites were stained using anti-GFP antibodies (green) and anti-Pys25 antibodies (red). Nuclei were stained by DAPI (blue). Representative immunofluorescence assay images for parasites at each stage (upper panels) and corresponding bright field images (lower panels) are shown. Bars: 5 µm. **c** The number of CryPH positive bodies was counted in a total of 300 CryPH-GFP expressing parasites. As the development of sexual stage parasites progressed from zygote, retort, to mature ookinete, the number of cell bodies expressing CryPH increased. Color codes in the column indicates the number of round bodies per parasite

To further confirm CryPH localization, genetically modified *Py*XNL expressing GFP-tagged CryPH (CryPH-GFP) was generated (see Additional file 1: Figure S1a). Expression of GFP-tagged CryPH in ookinetes was confirmed by Western blotting using anti-CryPH antibodies (Fig. 3a, left and middle panels). IFA with anti-GFP antibodies confirmed the localization profile of CryPH in zygotes and ookinetes (Fig. 3b). In addition, the change in the number of round bodies at each developmental stage from zygote to ookinete was examined using CryPH-GFP parasites. One CryPH-positive round body was observed in 98% of zygotes. In the course of parasite development, the number of round bodies in the cytoplasm increased; specifically, 70% and 28% of mature ookinetes contained two and three round bodies in the cytoplasm of each parasite, respectively (Fig. 3c).

### CryPH localizes to crystalloid bodies in ookinetes

**Fig. 4:**
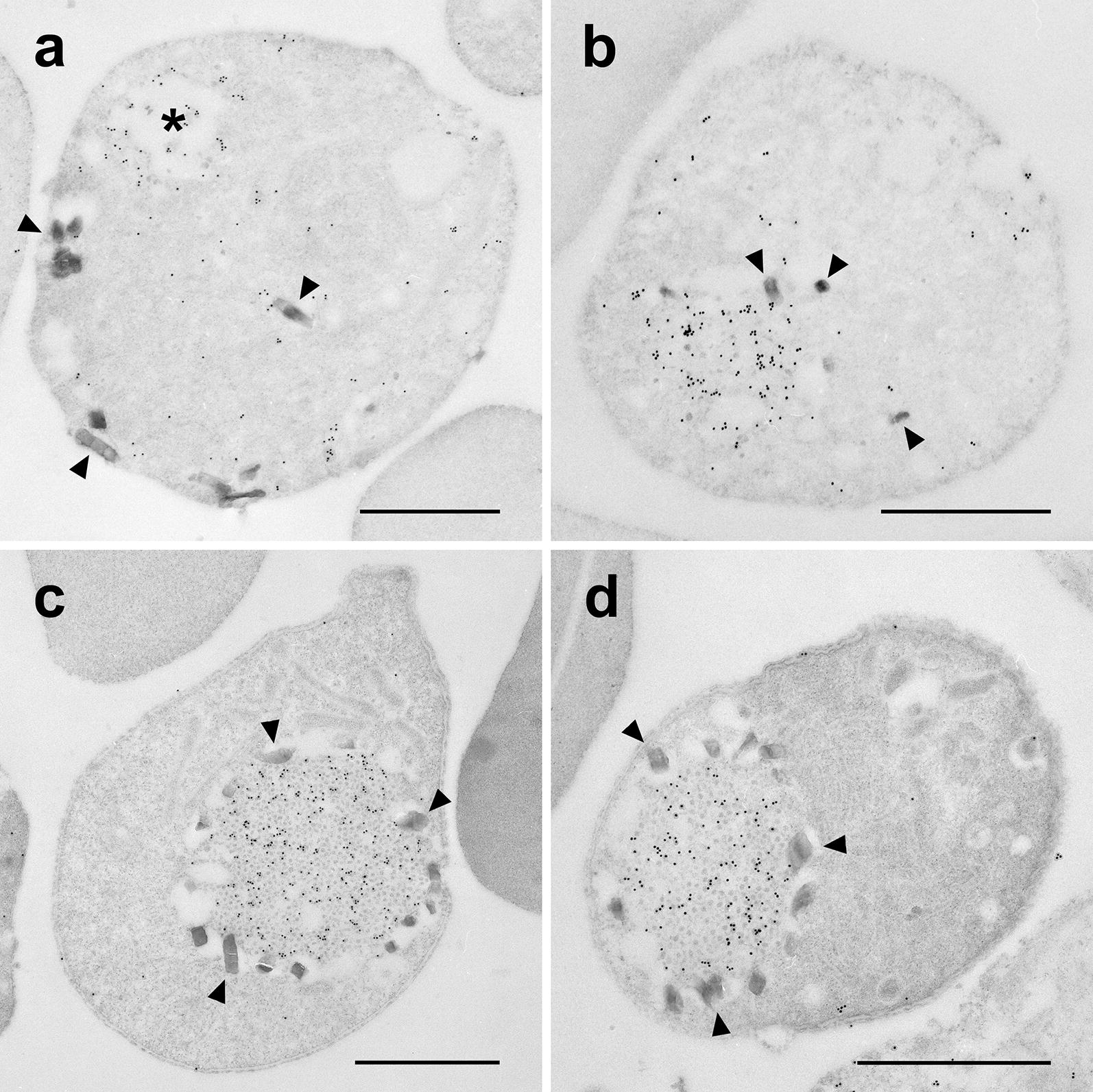
Detailed localization analysis of CryPH by immunoelectron microscopy. **a** Gold particles indicating the localization of CryPH were observed diffusely throughout the cytoplasm whereas some particles accumulated around the vacuole (*) in an early zygote. **b** Deposition of CryPH was observed when particle formation began in crystalloid bodies in zygotes. **c**, **d** In early retorts to mature ookinetes, CryPH localized to crystalloid bodies surrounded by malaria pigment. Arrowheads, malaria pigment. Bars: 1 µm

IEM was performed to determine the precise subcellular localization of CryPH in zygotes and ookinetes. Gold particles showing the localization of CryPH were observed scattered in the vacuolar space of the cytoplasm in the female gamete or in the early stage zygote (Fig. 4a). In zygotes, small high-density particles, characteristic of crystalloid bodies, appeared but they were not well aligned and malaria pigments (arrowheads) surrounding crystalloid bodies were scarce (Fig. 4b). In developing/mature ookinetes, CryPH signals were observed to accumulate in crystalloid bodies surrounded by malaria pigments (Fig. 4c, d), demonstrating that CryPH is a novel crystalloid body protein.

### CryPH is dispensable for ookinete and sporozoite invasive ability

**Fig. 5:**
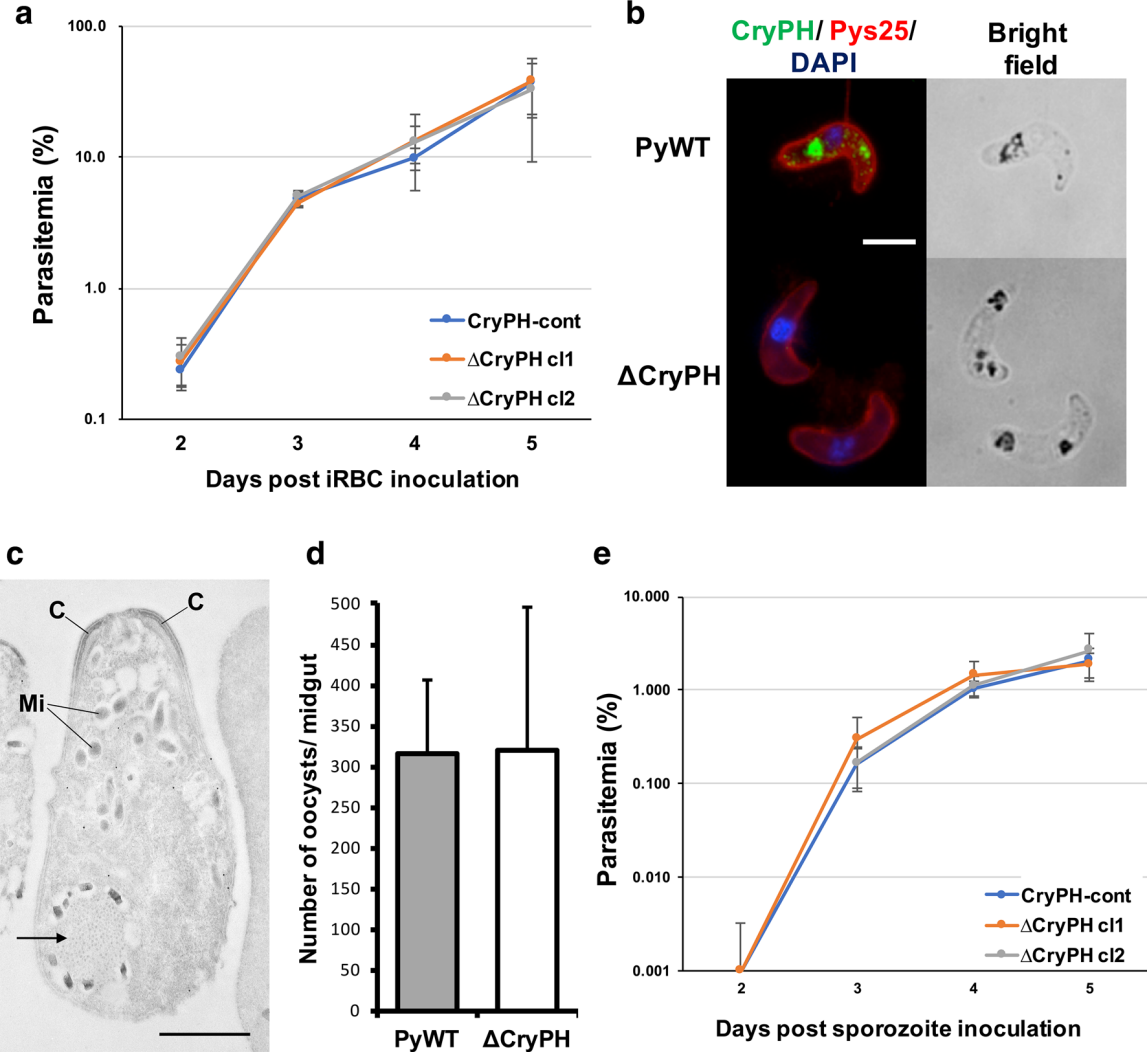
CryPH is dispensable for ookinete and sporozoite formation and their infectivity. **a** Erythrocytes (1 × 10^5^) infected with CryPH-cont or ∆CryPH (cl1 and cl2) parasites were inoculated intravenously into 4-week-old female ICR mice pretreated with phenylhydrazine. Parasitemias were examined daily by Giemsa staining. Parasitemia data from five mice are presented as the mean ± standard deviation. **b** Immunofluorescence assay of *Py*WT and ΔCryPH using anti-CryPH antibodies (green) and anti-Pys25 antibodies (red). Nuclei were stained by DAPI (blue). Bar: 5 µm. **c** Immunoelectron microscopy image of ΔCryPH showing normal ookinete apical structure. A crystalloid body (arrow) with a normal structure surrounded by malaria pigments is shown, but gold particles indicating CryPH localization were not observed. C: electron dense collar. Mi: microneme. Bar: 1 µm. **d** The number of oocysts formed on the mosquito midgut. Mosquitoes (PyWT, *n *= 20, ΔCryPH, *n *= 15) were dissected 10 days after they were fed with infected blood. **e** Sporozoites (1 × 10^4^) collected from salivary glands of CryPH-cont or ∆CryPH (cl1 and cl2) infected mosquitoes were inoculated intravenously into female ICR mice. Parasitemias were examined daily by Giemsa staining. Parasitemia data from five mice are presented as the mean ± standard deviation. No significant difference was observed in the infectivity between CryPH-cont and ∆CryPH sporozoites

To investigate the function of CryPH in zygotes and ookinetes, we generated *PyCryPH*-disrupted parasites (ΔCryPH) by homologous recombination to replace the endogenous *CryPH* locus with a human DHFR expression cassette as a selectable marker (see Additional file 1: Figure S1b). As a control, a human DHFR expression cassette was inserted at the same locus without changing CryPH expression (Additional file 1: Figure S1c, CryPH-cont). Two clones (ΔCryPH cl1 and ΔCryPH cl2), derived from independent transfections, and a CryPH-cont clone were successfully isolated. Successful integration of the selectable marker into the *CryPH* locus was confirmed by genotyping PCR (Additional file 1: Figure S1d). The ΔCryPH parasites at the intra-erythrocytic stage proliferated as efficiently as control parasites (CryPH-cont) by inoculation of parasite infected erythrocytes into mice, indicating that CryPH has no essential role in the blood-stage parasite development, as expected from its expression profile (Fig. 5a). After O/N culturing of infected erythrocytes, ΔCryPH developed into morphologically normal ookinetes (Fig. 5b). The absence of CryPH protein in ΔCryPH ookinetes was confirmed by Western blotting (Fig. 3a, right panels) and IFA (Fig. 5b, lower panels). To evaluate the morphology of ΔCryPH ookinetes in detail, electron microscopy was performed. Immunoelectron micrographs show that ΔCryPH ookinetes have a normal apical structure, micronemes, and crystalloid bodies surrounded by malaria pigments (Fig. 5c). This finding demonstrates that CryPH is dispensable for ookinete maturation including the formation of crystalloid bodies. The ability of ΔCryPH ookinetes to penetrate through the epithelial cells of mosquito midguts was unperturbed as the number of oocysts formed on the midgut wall at 10 days post-feeding was comparable in PyWT (315 ± 92; *n* = 20) and ΔCryPH (320 ± 176; *n* = 15) parasite lines (Fig. 5d). The effect of CryPH disruption on sporozoite formation and maturation was then examined to assess the ability to invade salivary glands. The average number of sporozoites residing in the salivary glands per mosquito were 33,800 (*n* = 23) and 19,100 (*n* = 21) in PyWT and ΔCryPH, respectively, in experiment 1; and 6900 (*n* = 18) and 6700 (*n* = 15) in PyWT and ΔCryPH, respectively, in experiment 2. Sporozoite transmission ability to mice was examined by inoculation of 10,000 sporozoites collected from salivary glands of CryPH-cont or ∆CryPH infected mosquitoes. As shown in Fig. 5e, no significant difference was detected in parasitaemias of CryPH-cont or ∆CryPH sporozoite inoculated mice, demonstrating that CryPH does not affect sporozoite infectivity. Taken together, these data suggest that CryPH is dispensable for the formation of ookinetes and sporozoites having complete invasive ability.

## Discussion

Here, CryPH was identified as being expressed in zygotes and ookinetes, and localized to crystalloid bodies. Structure prediction demonstrated that CryPH orthologues in *Plasmodium*, *Babesia*, *Theileria*, *and Cryptosporidium* have a PH domain with an N-terminal signal peptide. Recent comprehensive transcriptome and proteome analyses suggested that orthologues with the same structural feature exist in *Toxoplasma*.

The in silico screening used here to select secreted or membrane anchored-proteins expressed predominantly in the sexual-stage parasites also included two more PH domain-containing proteins, CryPH-p, encoded by a gene adjacent to CryPH, and PH. The orthologues of the PH domain-containing proteins are widely conserved across the orders in the phylum Apicomplexa, including *Plasmodium*, *Babesia*, *Theileria*, *Cryptosporidium*, and *Toxoplasma*, suggesting that they may have important roles common to these parasites. The PH domain, found in a wide range of proteins, binds phosphatidylinositol lipids and proteins such as the subunits of heterotrimeric G proteins and protein kinase C [59–61]. Through these interactions, the PH domain recruits proteins to different membranes, thereby targeting them to the proper cellular compartments [62], or interacts with the appropriate components of the signal transduction pathway [63–65]. IEM observation revealed that CryPH starts accumulating before the typical crystalloid body structure appears (Fig. 4b), raising the possibility that CryPH might be involved in crystalloid body formation by transporting proteins to the vesicles of crystalloid bodies in *Plasmodium*. Since CryPH orthologues are found in *Babesia*, *Theileria*, and possibly in *Toxoplasma*, in which crystalloid bodies have not been reported, another possibility is that CryPH may have more generic roles such as protein trafficking to target organelles.

The crystalloid body is a unique honeycomb-like structure observed in the cytoplasm of *Plasmodium* ookinetes and young oocysts, which has been suggested as a reservoir of proteins required for sporozoite formation. It has been demonstrated that LAP/CCp family proteins with characteristic LCCL domains are localized to crystalloid bodies in *P. berghei* and *P. falciparum* [26, 66]. Disruption of the LAP family proteins in *P. berghei* demonstrated that they are required for crystalloid body formation and subsequent sporozoite maturation inside oocysts, strongly supporting the hypothesis that crystalloid bodies contain proteins and/or nutrients required for parasite transmission (summarized in Table 1) [14, 67, 68]. However, the molecular mechanisms of crystalloid body formation and mode of action of LAP family proteins remain to be elucidated. The fact that all crystalloid body proteins, including CryPH, contain N-terminal signal peptides suggests that protein trafficking to the crystalloid bodies is dependent on the endoplasmic reticulum. By using targeted C*ryPH* gene-disruption it was revealed that, unlike LAP family proteins, CryPH is dispensable for parasite transmission via mosquitoes, that includes crystalloid body formation in ookinetes and sporozoite formation with the ability to invade salivary glands. Despite this, given that CryPH contains a PH domain in the secondary structure, and accumulates in vesicles prior to the typical crystalloid body formation implies that CryPH is involved in crystalloid formation. CryPH-p may compensate the role of CryPH in disrupted parasites, since their expression profile is similar. Localization analysis of CryPH-p and generation of double-knockout parasites will reveal their contribution for crystalloid formation. Future identification of proteins/molecules interacting with the PH domain of CryPH, for example by BioID technology, will likely provide clues to elucidate the biology of crystalloid body formation.

## Conclusions

In this study CryPH was described as a PH domain-containing protein, which specifically localized to crystalloid bodies in ookinetes of *P. yoelii*. This protein does not share the multi-domain architectures characteristic of the LAP/CCp family proteins which are also localized in crystalloid bodies. CryPH is dispensable for erythrocytic- and mosquito-stage parasite development, as demonstrated by the observation that genetically modified *CryPH*-deficient *P. yoelii* normally developed into ookinetes, oocysts, sporozoites as well as intra-erythrocytic-stage parasites.

## Additional file

**Additional file 1: Figure S1.** Construction of transgenic parasites with CryPH gene modifications. (a) Schematic representation of the generation of GFP-tagged CryPH expressing parasites. The native *PyCryPH* gene locus was replaced with a coding sequence of CryPH tagged by GFP at the C-terminal by double-crossover homologous recombination. The vector contains two homologous regions, the coding region of the C-terminal of CryPH (striped orange box, CryPH-C), which is connected in frame to GFP coding sequence (green box, GFP), and the 3′-UTR of CryPH (grid orange box, CryPH-3′). For selection of DNA integrated parasites, human DHFR coding sequence (dotted green box, hDHFR) is inserted between them. The resulting integrated locus is shown in the bottom panel. (**b**) Schematic representation of the targeted gene disruption of *CryPH*. The *CryPH-*coding region in the genome is replaced with the human *DHFR* expression cassette (dotted green box) by homologous recombination at the sites corresponding to the 5′- and 3′-UTR of *CryPH* (grid orange box). (**c**) Generation of CryPH-control (CryPH-cont) parasites. By homologous recombination, human *DHFR* expression cassette (dotted green box) is inserted next to the *CryPH* gene with the transgenic vector containing two homologous recombination sites corresponding to CryPH-C and 3′-UTR of *CryPH* (striped and grid orange boxes). Parasites with integrated DNA (bottom panel) were selected by drug treatment. **(d)** PCR genotyping of transgenic parasites. Correct DNA insertion into the *PyCryPH* locus of ∆CryPH (cl1 and cl2) and CryPH-cont transgenic parasites was confirmed by PCR using specific primer sets. The amplicons were diagnostic for: lane 1, integrated form; lane 2, episomal form; lane 3, wild-type. The expected sizes of the amplified fragments (lanes 1–3) were 1,000 bp, 1,235 bp, and 1,030 bp, respectively. Episomal form was not detected in any clones. **Figure S2.** Alignment of amino acid sequences of CryPH orthologues in apicomplexan parasites. Amino acid sequences of CryPH orthologues in *Plasmodium yoelii* (PY17X_0705200), *Toxoplasma gondii* (TGME49_219160), *Theileria equi* (BEWA_008110), *Babesia microti* (BMR1_02g03021), and *Cryptosporidium meleagridis* (CmeUKMEL1_06995) are aligned using CLUSTALW algorithm (https://npsa-prabi.ibcp.fr/cgi-bin/npsa_automat.pl?page=npsa_clustalw.html). Underbars indicate the signal peptide of each sequence. Strand-specific RNA-seq data suggested that translation of predicted *Toxoplasma* CryPH may start from the internal methionine (marked in orange). Approximately 3% of residues including all cysteine residues are conserved among CryPH orthologues. Similarity is higher in the PH domain predicted by the secondary structure of *Py*CryPH, indicated by a box. **Figure S3.** Molecular phylogenetic analysis by the Maximum Likelihood method. The evolutionary history was inferred using the Maximum Likelihood method [69]. The initial tree for the heuristic search was obtained automatically by applying Neighbor-Join and BioNJ algorithms to a matrix of pairwise distances estimated using a JTT model, and then selecting the topology with superior log likelihood value. All positions containing gaps and missing data were eliminated. Evolutional analyses were conducted in MEGA7 [70].

## Abbreviations

CryPH: crystalloid body specific protein containing PH domain
DAPI: 4′,6-diamidino-2-phenylindole
DHFR: dihydrofolate reductase gene
HRP: horseradish peroxidase
LAP: LCCL lectin adhesive-like proteins
PV: parasitophorous vacuole
SDS-PAGE: sodium dodecyl sulphate polyacrylamide gel electrophoresis
