# Supplementary material for: Identification of a PH domain-containing protein which is localized to crystalloid bodies of Plasmodium ookinetes
Source: Malar J. 2018 Dec 13;17:466. doi: 10.1186/s12936-018-2617-6 (PMC6291999; doi:10.1186/s12936-018-2617-6)
Supplement: Supplementary file 1 — Additional file 1: Figure S1. Construction of transgenic parasites with CryPH gene modifications. (a) Schematic representation of the generation of GFP-tagged CryPH expressing parasites. The native PyCryPH gene locus was replaced with a coding sequence of CryPH tagged by GFP at the C-terminal by double-crossover homologous recombination. The vector contains two homologous regions, the coding region of the C-terminal of CryPH (striped orange box, CryPH-C), which is connected in frame to GFP coding sequence (green box, GFP), and the 3′-UTR of CryPH (grid orange box, CryPH-3′). For selection of DNA integrated parasites, human DHFR coding sequence (dotted green box, hDHFR) is inserted between them. The resulting integrated locus is shown in the bottom panel. (b) Schematic representation of the targeted gene disruption of CryPH. The CryPH-coding region in the genome is replaced with the human DHFR expression cassette (dotted green box) by homologous recombination at the sites corresponding to the 5′- and 3′-UTR of CryPH (grid orange box). (c) Generation of CryPH-control (CryPH-cont) parasites. By homologous recombination, human DHFR expression cassette (dotted green box) is inserted next to the CryPH gene with the transgenic vector containing two homologous recombination sites corresponding to CryPH-C and 3′-UTR of CryPH (striped and grid orange boxes). Parasites with integrated DNA (bottom panel) were selected by drug treatment. (d) PCR genotyping of transgenic parasites. Correct DNA insertion into the PyCryPH locus of ∆CryPH (cl1 and cl2) and CryPH-cont transgenic parasites was confirmed by PCR using specific primer sets. The amplicons were diagnostic for: lane 1, integrated form; lane 2, episomal form; lane 3, wild-type. The expected sizes of the amplified fragments (lanes 1–3) were 1,000 bp, 1,235 bp, and 1,030 bp, respectively. Episomal form was not detected in any clones. Figure S2. Alignment of amino acid sequences of CryPH orthologues in apicomplexan parasites. A [file 12936_2018_2617_MOESM1_ESM.pdf]

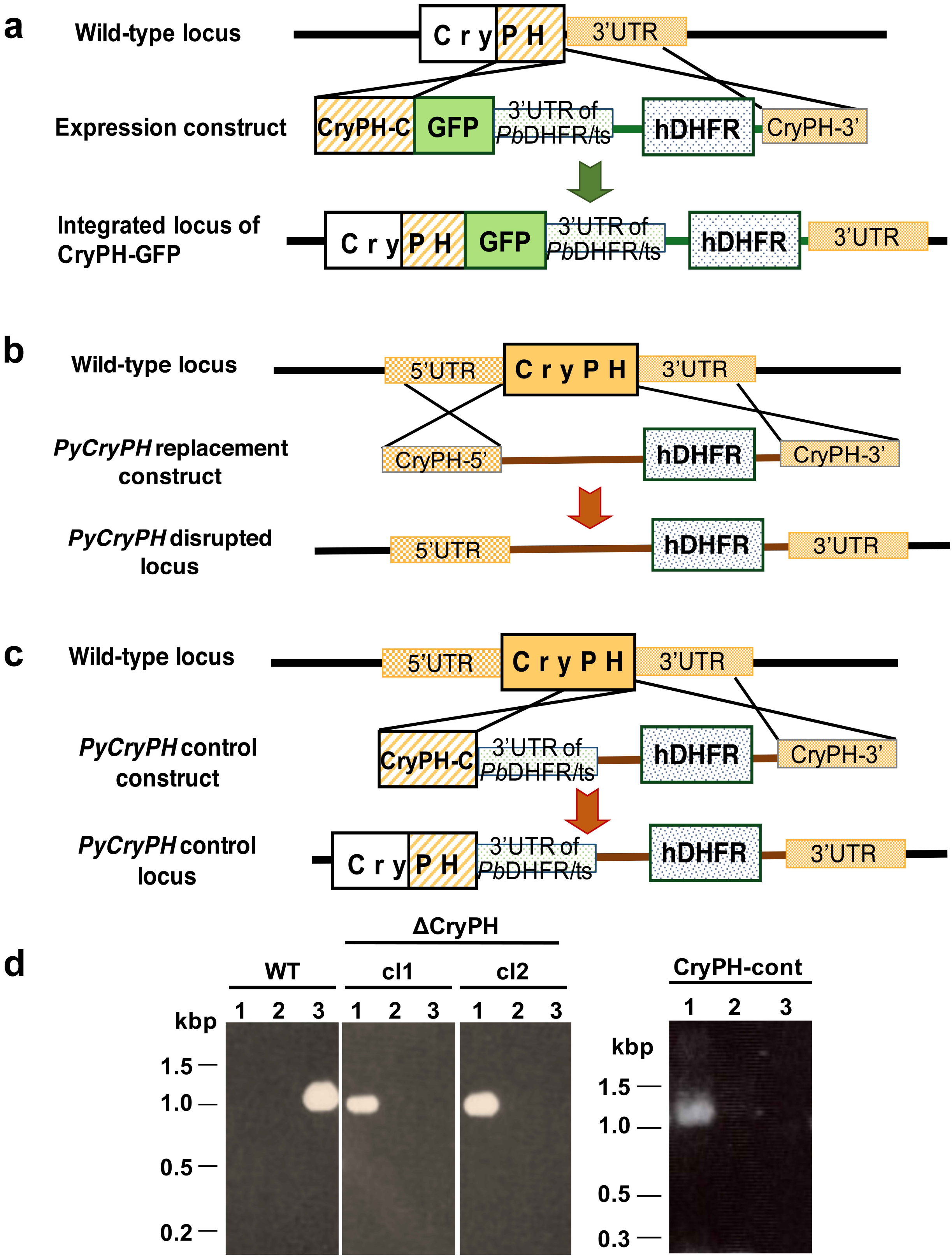
a
Wild-type locus
C r y P H
3'UTR
Expression construct
CryPH-C
GFP
3'UTR of PbDHFR/ts
hDHFR
CryPH-3'
Integrated locus of CryPH-GFP
C r y P H
GFP
3'UTR of PbDHFR/ts
hDHFR
3'UTR
b
Wild-type locus
5'UTR
C r y P H
3'UTR
PyCryPH replacement construct
CryPH-5'
hDHFR
CryPH-3'
PyCryPH disrupted locus
5'UTR
hDHFR
3'UTR
c
Wild-type locus
5'UTR
C r y P H
3'UTR
PyCryPH control construct
CryPH-C
3'UTR of PbDHFR/ts
hDHFR
CryPH-3'
PyCryPH control locus
C r y P H
3'UTR of PbDHFR/ts
hDHFR
3'UTR
d
ΔCryPH
WT
cl1
cl2
1 2 3 1 2 3 1 2 3
kbp
1.5
1.0
0.5
0.2
CryPH-cont
1 2 3
kbp
1.5
1.0
0.5
0.3

```
                        10        20        30        40        50        60        70        80        90       100
Toxoplasma      MLLITFPTPDSRTPTQVCFLVPHCPAKNTAS-GFKLRLSLGQETMKVSATASASCIAVLALVACRSEASPAFL--GSRGLGSRFEQPQTVEKEVEDAEEE
Theileria       --------------------------------------------MKIFDHTFLFYVVLYCLPNFICYT--HCL--NTIQNTNKLDHTLAIIKD----KDP
Babesia         -------------------------------------------------------MYLVTLITCVLWN----------PLHTNSKSVTDIENN-------
Plasmodium      --------------------MKVFVSISFVCLIAFCRGHKNFSRRNNYLQYLRSQTFMQEKPKLTNFNGNYSE--DITEFDIHEDDEYNNLKN----DDT
Cryptosporidium ------------------MKMRIFFLFNLLSVAGLSQYAHGRLTRSTSDFGLLEPQFLPGTITTLIQTARKIIGANVEPVSVIGEGKSQISQK--DSEKL
                                                                         :                          .      :.

                       110       120       130       140       150       160       170       180       190       200
Toxoplasma      LKTAEDDAALEEKNLESEISYRDSLRSQEQRDESAAKGMINVDAEAPGCSVETTGDMDVATDPTDVLTTVPMSISISKRHLSVNYKGRELKP--------
Theileria       LEEAEISEAYNIHSFEDEIKNKELLDTGSKND-------IVFSHKLSGCDTLRDDIIEVAVDPIDILKTQEFICGVTPNELRLYFKDRNNKSSNTLKKGK
Babesia         ---LID--TYRVNSLSDELRNSDFLDSGEFRIR------PLTLHKTTGCNAQMRGDLEAAIETLDMMRTQTFLAELSNSSFTLSYKGNKDKL--------
Plasmodium      KDTSNDKDMKENNKLEFKNLDKELNTIEVNNPDS-----INIMGSGKECSVNEKGELDVSINSQDIFNLIKYMVEITSN--SIIIKDIKNSN--------
Cryptosporidium YSEAVSSAVSVYQKAMNDATNNKLKQRDVITSMP-----VVDSPKDPVCSVMISGELALALDPRNIFDVDLLGVKITKDDLLFMTDLKNPES--------
                            :.   .    .                     .   *..   . :  : :. :::        ::     .  .  : .

                       210       220       230       240       250       260       270       280       290       300
Toxoplasma      -VMKIPLLEIKTPINTLARSRRCFRLFHRTKPLVFCADDTAARDEWIANIYKAVFCINSGNLLTPVQQKAKRE--KGESVPLPSKSTKVQRWIKEIARQE
Theileria       LFSRFSLNAIVTPLETIKSSRECFRLFYKSEPLVFCGKDAKARDLWMTSILKAKFCHYAHTNLNPAHKPETG----TLPQSLPKFSTKAERLKSRLLDKI
Babesia         -FGKFYLPKIHTPLETIVSSRTCWRLKYNENPLILCAKNTNQRDAWMSAIIKAIYCNAAGKSIGNDNAAKDA----TNKNELDKFELPKHSIVDKIKHKV
Plasmodium      VVKELSYDHIKLPIETIEETRECWSIKFNKEKIIFCEKNKQNRDNWVKDILKALFCYNTNNLTIENNQKVYK-----QKSDIPKHSTVNERIQNLKETLT
Cryptosporidium LVRSFSLNKIEVPLESVQNSRKCFRMYFDGSPVVMCAKSDEERNEMMNKLTEAIFCKNSGITFSKNTGTIENTGLSDSSLGVPALTTKDLRWIEQIAKKQ
                 .  :    *  *::::  :* *: : .  . :::* ..   *:  :  : :* :*  :                      :           .

                       310       320       330       340       350       360       370       380
Toxoplasma      AEALKEVQEGEM--MHRKKKNVVEEVEIVGGSAGAPPKVTVNGDELAFSHAGQREQLMNANNQEF---------------
Theileria       NDDVELP-------LHDNKITNVDIKNILTG----EPQIFLNGDEVIQNDTKTKTGQTS---------------------
Babesia         ATVHENIP------THDSGLTEIDIKNLLSG----SPLITVDGEEFPQTDHLTKNVDKNLEGTKPSEIGIERAIKPHGVM
Plasmodium      NDKNNFDKQ-----KSTKHNNNIVISNLKNS----NPNISLK--------------------------------------
Cryptosporidium IQMAGNVQSNVVNWLKSKGNQHVVVKDSLGFN----PQITVNGEKVV---------------------------------
                                 .    :   :         * : :.
```

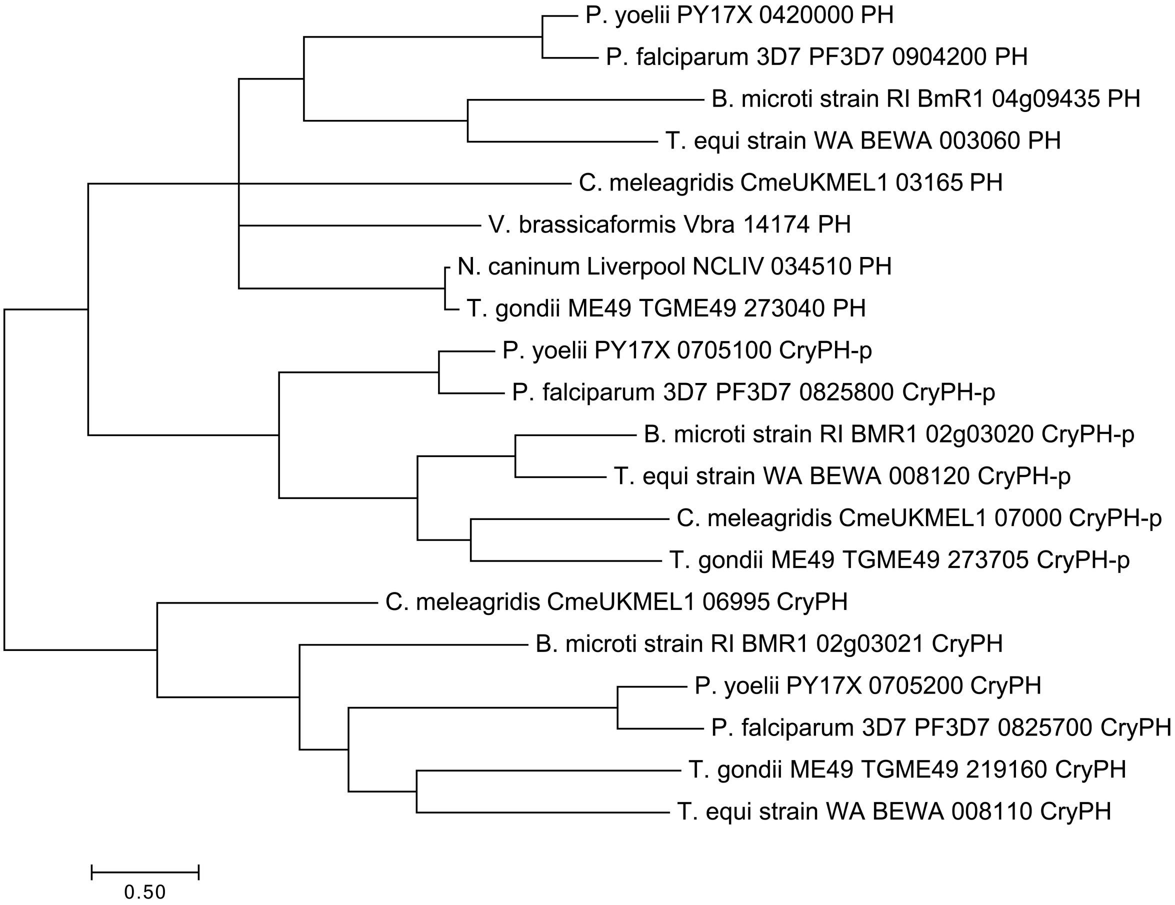
P. yoelii PY17X 0420000 PH
P. falciparum 3D7 PF3D7 0904200 PH
B. microti strain RI BmR1 04g09435 PH
T. equi strain WA BEWA 003060 PH
C. meleagridis CmeUKMEL1 03165 PH
V. brassicaformis Vbra 14174 PH
N. caninum Liverpool NCLIV 034510 PH
T. gondii ME49 TGME49 273040 PH
P. yoelii PY17X 0705100 CryPH-p
P. falciparum 3D7 PF3D7 0825800 CryPH-p
B. microti strain RI BMR1 02g03020 CryPH-p
T. equi strain WA BEWA 008120 CryPH-p
C. meleagridis CmeUKMEL1 07000 CryPH-p
T. gondii ME49 TGME49 273705 CryPH-p
C. meleagridis CmeUKMEL1 06995 CryPH
B. microti strain RI BMR1 02g03021 CryPH
P. yoelii PY17X 0705200 CryPH
P. falciparum 3D7 PF3D7 0825700 CryPH
T. gondii ME49 TGME49 219160 CryPH
T. equi strain WA BEWA 008110 CryPH
0.50
